# Supervised Machine Learning Algorithms for Fitness-Based Cardiometabolic Risk Classification in Adolescents

**DOI:** 10.3390/sports13080273

**Published:** 2025-08-18

**Authors:** Rodrigo Yáñez-Sepúlveda, Rodrigo Olivares, Pablo Olivares, Juan Pablo Zavala-Crichton, Claudio Hinojosa-Torres, Frano Giakoni-Ramírez, Josivaldo de Souza-Lima, Matías Monsalves-Álvarez, Marcelo Tuesta, Jacqueline Páez-Herrera, Jorge Olivares-Arancibia, Tomás Reyes-Amigo, Guillermo Cortés-Roco, Juan Hurtado-Almonacid, Eduardo Guzmán-Muñoz, Nicole Aguilera-Martínez, José Francisco López-Gil, Vicente Javier Clemente-Suárez

**Affiliations:** 1 Faculty Education and Social Sciences, Universidad Andres Bello, Viña del Mar 2520000, Chile; rodrigo.yanez.s@unab.cl (R.Y.-S.); jzavala@unab.cl (J.P.Z.-C.); claudio.hinojosa@unab.cl (C.H.-T.); frano.giakoni@unab.cl (F.G.-R.); josivaldo.desouza@unab.cl (J.d.S.-L.); 2 Escuela de Ingeniería Informática, Universidad de Valparaíso, Valparaíso 2362905, Chile; rodrigo.olivares@uv.cl (R.O.); pablo.olivareszu@uv.cl (P.O.); 3 Exercise and Rehabilitation Sciences Laboratory, School of Physical Therapy, Faculty of Rehabilitation Sciences, Universidad Andres Bello, Santiago 7610196, Chile; matias.monsalves@unab.cl (M.M.-Á.); marcelo.tuesta@unab.cl (M.T.); 4 Grupo eFidac, Escuela de Educación Física, Pontificia Universidad Católica de Valparaíso, Valparaíso 2340000, Chile; jacqueline.paez@pucv.cl (J.P.-H.); juan.hurtado@pucv.cl (J.H.-A.); 5 Grupo AFySE, Investigación en Actividad Fìsica y Salud Escolar, Escuela de Pedagogía en Educación Fìsica, Facultad de Educación, Universidad de Las Américas, Santiago 7500000, Chile; jolivares@udla.cl; 6 Observatorio de Ciencias de la Actividad Física (OCAF), Departamento de Ciencias de la Actividad Física, Universidad de Playa Ancha, Valparaíso 2340000, Chile; tomas.reyes@upla.cl; 7 Facultad de Ciencias de la Vida, Universidad Viña del Mar, Viña del Mar 2520000, Chile; guillermo.cortes@uvm.cl; 8 Escuela de Kinesiología, Facultad de Salud, Universidad Santo Tomás, Talca 3460000, Chile; eguzmanm@santotomas.cl; 9 Escuela de Kinesiología, Facultad de Ciencias de la Salud, Universidad Autónoma de Chile, Talca 3460000, Chile; 10 Facultad Ciencias de la Salud, Universidad Católica del Maule, Talca 3460000, Chile; naguilera@ucm.cl; 11 School of Medicine, Universidad Espíritu Santo, Samborondón 092301, Ecuador; 12 Vicerrectoría de Investigación y Postgrado, Universidad de Los Lagos, Osorno 5290000, Chile; 13 Faculty of Medicine, Health and Sports, Universidad Europea de Madrid, Madrid 28670, Spain; vctxente@yahoo.es; 14 Grupo de Investigación en Cultura, Educación y Sociedad, Universidad de la Costa, Barranquilla 080002, Colombia

**Keywords:** gradient boosting, health, physical fitness, adolescent, predictive modeling

## Abstract

Background: Cardiometabolic risk in adolescents represents a growing public health concern that is closely linked to modifiable factors such as physical fitness. Traditional statistical approaches often fail to capture complex, nonlinear relationships among anthropometric and fitness-related variables. Objective: To develop and evaluate supervised machine learning algorithms, including artificial neural networks and ensemble methods, for classifying cardiometabolic risk levels among Chilean adolescents based on standardized physical fitness assessments. Methods: A cross-sectional analysis was conducted using a large representative sample of school-aged adolescents. Field-based physical fitness tests, such as cardiorespiratory fitness (in terms of estimated maximal oxygen consumption [VO_2max_]), muscular strength (push-ups), and explosive power (horizontal jump) testing, were used as input variables. A cardiometabolic risk index was derived using international criteria. Various supervised machine learning models were trained and compared regarding accuracy, F1 score, recall, and area under the receiver operating characteristic curve (AUC-ROC). Results: Among all the models tested, the gradient boosting classifier achieved the best overall performance, with an accuracy of 77.0%, an F1 score of 67.3%, and the highest AUC-ROC (0.601). These results indicate a strong balance between sensitivity and specificity in classifying adolescents at cardiometabolic risk. Horizontal jumps and push-ups emerged as the most influential predictive variables. Conclusions: Gradient boosting proved to be the most effective model for predicting cardiometabolic risk based on physical fitness data. This approach offers a practical, data-driven tool for early risk detection in adolescent populations and may support scalable screening efforts in educational and clinical settings.

## 1. Introduction

Physical fitness has been consistently linked to cardiometabolic risk in children and adolescents [1,2,3]. In particular, high cardiorespiratory fitness levels [4,5] and muscle strength [6,7] from an early age are associated with lower mortality and reduced risk factors for cardiovascular disease (CVD) [8]. Low muscle strength is associated with metabolic risk factors in children [9], and inverse associations have been observed between muscle fitness and inflammatory biomarkers [10,11]. While obesity-related variables may hypothetically mediate the association between fitness and cardiometabolic risk, the importance of fitness should not be overlooked. In this regard, improving fitness to maintain lower body fat may be crucial to achieving a healthier cardiometabolic profile [1]. This underpins a shift in approach to cardiometabolic risk assessment from an obesity-centered perspective to a multifactorial perspective that incorporates fitness and other risk factors [12,13,14]. There is now evidence showing that early-onset obesity is associated with an increased risk of metabolic syndrome [15,16], which is considered a clustering of cardiovascular and metabolic risk factors, such as abdominal obesity, hypertension, insulin resistance, elevated triglycerides (TGs), and low high-density lipoprotein (HDL) cholesterol. It has been observed in children and adolescents and tends to persist from childhood into adulthood [17,18]. Furthermore, CVDs are a relevant concern in pediatric populations, as atherosclerosis is a multifactorial condition characterized by a slow and progressive course. It primarily affects medium- and large-sized arteries and often manifests clinically through thrombotic events [19]. Although atherosclerosis exhibits clinically in middle and late adulthood, it is well known that it has a long asymptomatic phase of development, which starts in childhood, and in most cases, children have mild atherosclerotic vascular disorders, which can be avoided or reduced by adopting healthy lifestyle habits [20,21,22].

Data quantifying the burden of cardiometabolic risk factors in South American children (0–21 years) show that 12.2% have obesity, 21.9% have elevated waist circumference, 3.0% have elevated fasting blood glucose, 18.1% have elevated triglycerides, 29.6% have low HDL cholesterol, and 8.6% have high blood pressure [23]. When the levels of cardiometabolic risk according to metabolic syndrome among Chilean adolescents and international data are compared, 9.5% of adolescents in Chile present with metabolic syndrome [24,25]. The new physical activity recommendations state that children and adolescents should perform at least an average of 60 min per day of moderate-to-vigorous-intensity physical activity, mainly aerobics, throughout the week [26].

Following the Coronavirus Disease 2019 (COVID-19) pandemic, various factors have reduced adherence to these recommendations, leading instead to the development of habits characterized by increased use of digital devices. Among adolescents aged 11 to 17 years, the daily time spent on digital devices has risen by 0.9 h [27]. This shift has had negative implications for the health of children and adolescents, particularly considering that sedentary behavior may act as an independent risk factor for physical inactivity [28]. Consequently, there is a growing need to closely monitor sedentary time within school populations [29].

In addition, physical inactivity is a global public health problem that significantly impacts the world’s population. In Chile, data from different surveys suggest that physical activity behavior is low [30], and the results of Chile’s 2022 Report Card [31], like its previous versions, show persistently low grades for most indicators [32]. Therefore, in Chile, approximately one out of five children and youth are physically inactive, meaning Chile is among the world’s most inactive countries [33].

Although numerous food and nutrition initiatives have been implemented in Chile since the early twentieth century, such as the food labeling law, restrictions on unhealthy food advertising, and the promotion of healthy kiosks, the prevalence of excess weight among school children remains high, with 27% having overweight and 23.9% having obesity, totaling 50.9% of school children [34]. However, no existing methods enable the use of machine learning algorithms to identify cardiometabolic risk from physical fitness in this group [35]. According to recent data from the Global Burden of Disease (GBD) [36], the prevalence of both overweight and obesity increased significantly in all regions of the world between 1990 and 2021. The increase in obesity is expected to continue in all populations in all regions of the world, emphasizing the need for more substantial and more targeted measures to address this crisis, as obesity is one of the leading preventable health risks, and this will continue to be the case in the future. It represents an unprecedented threat of premature illness and death locally, nationally, and globally [37].

While cardiometabolic risk assessment systems exist, there are no methods that allow for the use of machine learning algorithms to identify cardiometabolic risk from physical fitness in this group, and few studies have employed machine learning models to predict cardiovascular risk [38,39,40]. The increasing advancement of artificial intelligence and automation offers an excellent opportunity to develop models that allow for the processing of large databases to better understand the phenomena and the interaction of physical fitness with health in adolescents. Most previous studies have focused on the association of a single risk factor, and studies that consider the impact of more than one risk factor on CVD use different forms of regression or multivariate analysis and assume that risk factors are related to CVD, with this relation following a linear pattern. Moreover, some studies do not consider other behavioral and lifestyle factors as predictors [38].

Given the increasing burden of cardiometabolic disorders among youth populations in Latin America, the present study contributes to the advancement of data-driven strategies by demonstrating the potential of machine learning models to process large-scale, representative datasets. These models can generate high-quality, interpretable insights to inform evidence-based public health policies targeting obesity prevention and the reduction of sedentary behaviors. Such approaches are crucial for improving population health outcomes and quality of life across the lifespan. In this context, the objective of this study was to develop and evaluate supervised machine learning algorithms, including artificial neural networks and ensemble methods, for classifying cardiometabolic risk levels among Chilean adolescents based on standardized physical fitness assessments.

## 2. Materials and Methods

### 2.1. Study Design

A cross-sectional observational study was conducted. The analysis was based on data from the national System for Measuring the Quality of Education (SIMCE Physical Education assessment) [41], which was administered to 8th-grade students. Based on these data, machine learning models were developed to classify cardiometabolic risk using physical fitness indicators as predictive variables. The manuscript was developed following the Strengthening the Reporting of Observational Studies in Epidemiology (STROBE) guidelines for cross-sectional studies [42], to promote transparency and ensure the reproducibility of the report.

### 2.2. Procedure

The data were obtained from the official repository of the Chilean Ministry of Education. An exhaustive process of data cleaning and variable standardization was carried out to ensure adequate preprocessing and modeling for the analyses. Data from the evaluation conducted during 2015 were included. Data were obtained via an information access application; the link to access the databases is available at https://informacionestadistica.agenciaeducacion.cl/#/bases (Accessed on 3 March 2025).

### 2.3. Participants

The study included a total of 7854 Chilean adolescents with a mean age of 15.9 years (3498 females and 4356 males), representing all regions of Chile. The sample was selected via stratified sampling by region, school type (public, subsidized, or private), and sex. The inclusion criteria required participants to complete all physical and anthropometric assessments of the SIMCE-EFI test [41]. Exclusion criteria included cases with critical missing data or medical conditions preventing physical activity. The dataset represents a nationally representative sample provided by the Agency for Educational Quality of the Ministry of Education of the Government of Chile. Although the SIMCE-EFI 2015 dataset comes from a nationally representative sample, based on stratification by region, type of establishment, and gender, the use of a single year of measurement may bring limitations in temporal generalization and does not necessarily capture interannual or contextual variations after 2015, such as the effects of the pandemic or changes in public policy.

### 2.4. Ethical Considerations

The present study was performed after the participants and guardians provided signed informed consent and assent. Sessions were held to detail the procedures and scope of the study prior to the evaluations. All the evaluations were performed in accordance with the recommendations of the Declaration of Helsinki for studies involving human beings [43], and the Council for International Organizations of Medical Sciences (CIOMS) standards were also followed. The original data collection obtained informed consent/assent and was approved by the Education Quality Agency (SIMCE-EFI Project). The present secondary analysis of anonymized data was deemed exempt from further review by the institutional ethics committee.

### 2.5. Procedures

#### 2.5.1. Physical Fitness

Three core components of health-related physical fitness were evaluated, following the standards established in the SIMCE-EFI protocol. Cardiorespiratory capacity was assessed with the 20-m shuttle run test (estimated VO_2max_). Upper-body muscular endurance was measured with the push-up test. Lower-limb explosive power was assessed with the horizontal jump. Abdominal strength/endurance was assessed with the 30-s sit-up test, following the SIMCE-EFI protocol [41].

#### 2.5.2. Anthropometric Variables

Anthropometric measurements were conducted in accordance with the standards of the World Health Organization [44] and the SIMCE protocol. The following measures were included. The height was measured with a portable stadiometer (precision ± 0.1 cm). Waist circumference was measured at the level of the umbilicus (precision ± 0.1 cm). Cardiometabolic risk was estimated using the waist-to-height ratio (WHtR).

#### 2.5.3. Cardiometabolic Risk

Cardiometabolic risk was estimated via the WHtR, with a threshold of ≥0.5 to indicate central obesity [45]. This approach has been widely recommended in longitudinal studies because of its sensitivity in detecting early risk of cardiovascular and metabolic diseases in school-aged populations [46,47]. The WHtR has been validated as a robust indicator of central fat distribution and metabolic risk and has shown greater predictive capacity than body mass index (BMI) in pediatric populations. The participants were categorized into two groups based on international cutoff points [48] (no risk: WHtR < 0.5; at risk: WHtR ≥ 0.5). Although WHtR represents a single component of cardiometabolic risk (specifically central obesity) it is a strong predictor of multiple cardiometabolic outcomes, including dyslipidemia, hypertension, and insulin resistance in pediatric populations [46,47]. Several studies have shown that WHtR has greater predictive power than BMI or waist circumference alone in identifying overall cardiometabolic risk, allowing for accessible and sensitive early detection in school or community settings [47,49]. In this study, WHtR was used as a validated, practical, and clinically significant indicator for the dichotomous stratification of cardiometabolic risk.

### 2.6. Statistical Analysis

The results of the continuous variables were presented as median and interquartile ranges (IQRs) due to the non-normal distribution of the data. These were presented according to sex and with the total sample. The program used for this analysis was jamovi (version 2.3.21).

### 2.7. Supervised Machine Learning Models

The objective of the model was to classify cardiometabolic risk levels based on physical fitness variables. Five supervised classification algorithms were applied, using estimated VO_2max_, horizontal jumps, and push-ups as predictors. Cardiometabolic risk was defined according to the WHtR, with values < 0.5 indicating normal risk and values ≥ 0.5 indicating high risk.

#### 2.7.1. Algorithm Analysis

Machine learning models were used for the analysis of the algorithms, and SHapley Additive exPlanations (SHAP) were also used to improve the interpretability of the results by contributing to model understanding and prediction, thus improving model transparency and reliability [50]. The machine learning analysis was performed in Jupyter Notebook (Version 7.1), and the Python programming language (version 3.13) was used to develop the codes. 

#### 2.7.2. Processing Models

A supervised classification analysis was developed to classify cardiometabolic risk (based on WHtR) using estimated VO_2max_, push-ups, and horizontal jump as predictors. The following predictor variables were used: estimated maximal oxygen consumption [VO_2max_], push-ups, and horizontal jumps. To control for the possible effect of demographic variables on risk classification, the supervised learning algorithms were adjusted for age and sex. Both variables were incorporated as explicit covariates in the model’s set of predictors, coded numerically. No prior stratified sampling was performed for these variables, as their direct inclusion as predictors allowed the models to capture potentially nonlinear relationships and complex interactions with physical condition variables. All the data were obtained from a national database of Chilean adolescents in the context of a standardized physical evaluation conducted by an agency ensuring the quality of education in Chile, part of the Ministry of Education in Chile.

#### 2.7.3. Data Preprocessing

Data preprocessing is a crucial step to ensure that machine learning models receive clean, consistent, and appropriate data, leading to reliable results. This process included the following specific steps:

##### Numeric Format Normalization

The original dataset contained numeric values with commas a decimal separator, a format common in Spanish-speaking countries. However, Python and most data science libraries, including scikit-learn, expect the use of a period (.) as the decimal separator. All commas were replaced by periods by string replacement techniques, allowing for subsequent accurate conversion of these text strings into numeric values.

##### Data Type Conversion

After normalizing the decimal separators, all relevant columns (predictor variables and the target variable) were explicitly transformed into numeric data types (float or int) via functions such as astype() in Pandas. This step is critical since machine learning models require numeric inputs to perform internal mathematical and statistical operations.

##### Handling Missing Data

Rows containing missing values were removed. This method was chosen for simplicity and robustness, ensuring that all models received complete datasets without the need to impute missing values, which could have introduced biases or altered the original data distribution.

##### Encoding Categorical Variables

The target variable (clasification WHtR), indicating cardiometabolic risk classified into different categories, was transformed into an integer. This transformation is essential for the proper functioning of supervised classification algorithms, which require clearly identifiable numeric categories (e.g., 0 and 1 represent low and high risk, respectively).

##### Dataset Splitting

The cleaned and transformed dataset was divided into two independent subsets.

Training Set (80%): This set was used to train and tune the internal parameters of supervised models.

Test Set (20%): This set was used to evaluate the predictive ability and generalized performance of the models.

The split was performed via the train_test_split() function from scikit-learn, with a fixed random seed (random_state = 42) to ensure consistency and reproducibility in future studies.

#### 2.7.4. Classification Models

Five widely used supervised machine learning algorithms were implemented to classify cardiometabolic risk. These machine learning approaches included gradient boosting (GB), a technique that builds decision trees sequentially to correct previous errors and is known for its high predictive performance on tabular data; logistic regression (LR), a linear model that estimates the probability of categorical outcomes through a logistic function, valued for its simplicity and interpretability; and K-NN, a nonparametric method based on the assumption that similar observations are located near each other, requiring no assumptions about data distribution. Support vector machines (SVM) with a linear kernel were applied, suitable for linearly separable problems. For feature importance, we additionally used the linear support vector classifier (LinearSVC, dual = False), a variant of SVM that provides direct coefficients to quantify each feature’s contribution. Finally, random forest (RF), an ensemble of decision trees based on bagging, was included for its ability to model complex nonlinear relationships.

#### 2.7.5. Best Algorithm Analysis

To identify the most effective model, several key performance metrics were evaluated, each capturing different aspects of classification quality. Accuracy was used to measure the overall proportion of correct predictions relative to the total number of predictions. Recall assessed the model’s ability to identify positive true positives, while the F1 score provided a balanced measure between precision and recall, particularly valuable in contexts with class imbalance. Additionally, the area under the receiver operating characteristic curve (AUC-ROC) curve was examined to evaluate the model’s overall capacity to distinguish between classes across all possible decision thresholds.

#### 2.7.6. Performance Evaluation

The models were evaluated exclusively on the test dataset to prevent overfitting bias. Accuracy, recall, and F1 score metrics were calculated via functions from the sklearn.metrics module, which applies a weighted average (average = ‘weighted’) to adequately account for possible class imbalances in the dataset.

Two approaches were used to evaluate variable importance, depending on the model type.

We used linear models (LR, SVM), with normalized absolute coefficients (coef_).

We also used tree-based models (RF, GB), with built-in feature importance methods (feature_importances_) reported as relative percentages.

GB stands out, particularly because of its balance across metrics, stability, and interpretability facilitated by SHAP analysis, which transparently illustrates how each variable contributes individually and globally to predictions.

This preprocessing procedure, combined with model evaluation and selection of the best-performing algorithm, resulted in promising outcomes for identifying cardiometabolic risk based on physical fitness tests in adolescents.

The machine learning models were trained using default hyperparameter values. This decision was methodologically justified as part of a baseline exploration strategy aimed at establishing a benchmark for future performance improvements. According to Probst [50], understanding a model’s performance under default settings is essential for evaluating the real benefits of later hyperparameter tuning (Table 1).

Regarding model validation, cross-validation was intentionally omitted in this exploratory phase. Instead, a single train–test split (80/20) was used with a fixed random seed to ensure reproducibility. This choice reflects a phase-based methodological approach, where rapid prototyping and feasibility assessment precede more rigorous validation in subsequent studies. The goal was to detect predictive signals and evaluate modeling viability, not to obtain final performance estimates.

To address class imbalance, random undersampling was applied to the majority class. This technique balances class distribution by randomly selecting a subset of samples from the majority class to match the size of the minority class. While this entails discarding a portion of data, thereby risking some loss of information, this approach was selected for its computational efficiency and compatibility with limited hardware resources. More advanced techniques such as synthetic minority over-sampling technique (SMOTE), though potentially superior, were not feasible given computational constraints [51].

## 3. Results

Table 2 shows that adolescents at higher risk (Level 2) had consistently lower values from the VO_2max_, horizontal jump, and push-up testing compared to their low-risk peers (Level 1), with this holding true in both sexes. For example, median VO_2max_ was 27.4 mL/kg/min (IQR: 26.4–28.9) in the risk group versus 28.9 (IQR: 27.4–30.6) in the non-risk group; horizontal jump reached 136.5 cm (IQR: 115.0–158.5) versus 140.7 (IQR: 124.0–172.0); and the median for push-ups were 13.0 repetitions (IQR: 7.0–19.0) versus 15.0 (IQR: 10.0–20.0), respectively. In addition, the median WHtR was notably higher in the at-risk group (median = 0.500 [IQR: 0.460–0.560]) than in the low-risk group (median = 0.430 [IQR: 0.410–0.450]), cementing it as a sensitive and practical marker. These results support the use of low-logistical-complexity physical field tests, such as the horizontal jump test, push-up testing, and the 20-meter test for estimating VO_2max_, as they are accessible tools for the early detection of cardiometabolic risk in school populations. On separating the results according to sex, it was observed that students classified at the lowest risk level had higher values in the physical fitness tests. 

Table 3 presents a comparison of the different classification algorithms in terms of accuracy, classification error, precision, recall, F1 score, AUC-ROC, and training time. The model with the highest F1 score was k-nearest neighbors (K-NN) (0.697); however, it exhibited a relatively low AUC-ROC (0.548), indicating limited discriminative power. GB demonstrated a competitive balance between accuracy (0.770) and F1 score (0.673) while also achieving the highest AUC-ROC among the tested models (0.601). LR showed the shortest training time. Although the SVM algorithm achieved similar performance metrics to LR, it required the longest training time (>8 s). All models yielded F1 scores in the range of 0.67 to 0.70, indicating that the classification task involves non-trivial class separation. The performance difference between GB and K-NN was minimal (Δ = 0.024), yet GB was favored due to its greater interpretability through SHAP and superior model stability. Therefore, GB was selected as the most suitable model for cardiometabolic risk classification in this study. Although the GB and LR models presented identical values for precision (0.598) and sensitivity (0.773), a slight difference was observed in the F1 score (0.673 vs. 0.674, respectively). This discrepancy is due to the calculation of the class-weighted F1 score, which incorporates the support or number of instances per class in the test set. In tasks with unbalanced classes, slight variations in the counts of true positives, false positives, and false negatives per class can generate minimal differences in the F1 score, even when the overall metrics match. Therefore, this difference is expected and does not imply a contradiction to the results.

**Figure 1:**
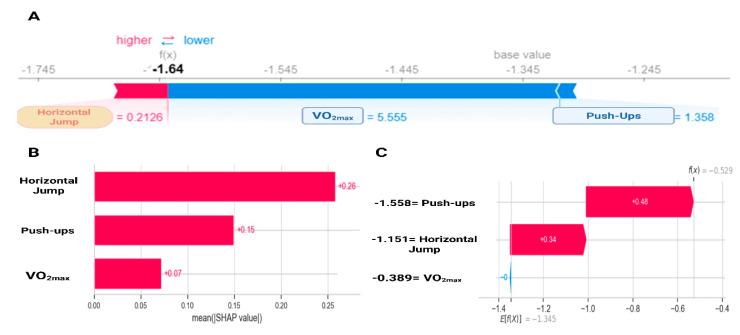
SHapley Additive exPlanations analysis for gradient boosting on the contribution of physical fitness variables to cardiometabolic risk. (**A**) Individual variable contribution according to SHAP; (**B**) Global variable importance according to SHAP; (**C**) Individual contribution in a specific case. VO_2max_: estimated maximal oxygen consumption.

Figure 1 shows how the different physical fitness components contribute to the probability of being classified as at risk (WHtR ≥ 0.5). Low VO_2max_ and push-up values are associated with positive SHAP values, meaning they increase the probability of belonging to the risk group. Conversely, higher values of these tests present negative SHAP values, lowering the predicted risk. Horizontal jump follows the same pattern, but with stronger global importance. The feature importance ranking (Figure 1B) confirms horizontal jump as the most relevant predictor (0.26, 26%), followed by push-ups (0.15, 15%) and VO_2max_ (0.07, 7%). This result mirrors the exploratory analysis and highlights horizontal jump as the single best predictor. An illustrative case (Figure 1C) further demonstrates the interpretability of the model: high values in horizontal jump (+0.34) and push-ups (+0.48) push the prediction toward the low-risk class (i.e., they result in negative SHAP values, decreasing the predicted probability of cardiometabolic risk), providing a transparent additive explanation.

**Figure 2:**
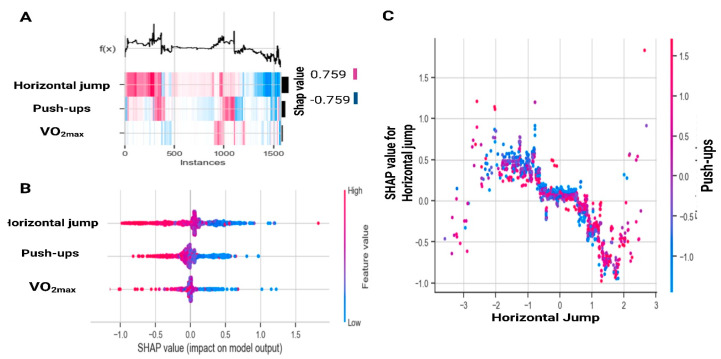
SHapley Additive exPlanations analysis for all instances based on gradient boosting. (**A**) SHapley Additive exPlanations values across instances; (**B**) SHapley Additive exPlanations value distribution by feature; (**C**) Interaction effects between Horizontal Jump and Push-ups. VO_2max_: estimated maximal oxygen consumption.

Figure 2 expands on these findings at the population level. Figure 2A displays SHAP values across all instances, where horizontal jump shows the greatest variability, push-ups contribute moderately, and VO_2max_ has a smaller impact. Figure 2B illustrates that high values of jump and push-ups (red, positive SHAP) reduce the probability of being classified as at risk, whereas low values (blue, negative SHAP) increase the likelihood of risk. Finally, Figure 2C shows the nonlinear relationship between jump distance and SHAP values, revealing a saturation effect beyond approximately 120 cm, along with a mild interaction with push-ups.

## 4. Discussion

This study analyzed supervised machine learning models to classify cardiometabolic risk using field-based fitness tests. Although machine learning models for cardiovascular risk prediction in youth already exist [38], they typically depend on clinical biomarkers, survey data, or self-reported indicators. The novelty of our contribution lies in its leveraging of exclusively field-based fitness tests, alongside minimal anthropometrics, to build a scalable screening approach for school settings, particularly relevant in Latin American contexts with limited access to laboratory measurements. The GB classifier showed the best overall performance, achieving an accuracy of 77.0%, an F1 score of 67.3%, and the highest AUC-ROC (0.601). These results show a strong balance between sensitivity and specificity in classifying adolescents at cardiometabolic risk. According to the results of the SHAP analysis for GB on the contribution of physical fitness variables to cardiometabolic risk, horizontal jumping had the most significant importance, push-ups had medium importance, and VO_2max_ had the least importance.

These results are consistent with the findings of Ortega et al. [6], who reported that performance on jumping tests is inversely related to cardiovascular risk markers in children and adolescents. This supports the notion that field-based muscular fitness assessments, particularly those involving lower-limb power, may serve as accessible and informative proxies for cardiometabolic health. Similarly, Delgado-Floody et al. [52] found that both the horizontal jump test and cardiorespiratory fitness were inversely associated with predictors of CVD risk in Chilean schoolchildren. These findings underscore the critical role of muscular fitness, encompassing strength, endurance, and explosive power, not only for enhancing physical performance but also as a modifiable determinant of metabolic health [53]. In fact, components of the metabolic syndrome, such as abdominal obesity, hypertension, and dyslipidemia, have been negatively associated with muscular strength in adolescents. This implies that interventions combining aerobic and resistance training are effective. Consequently, enhancing muscular fitness from an early age may be a key strategy for mitigating future CVD risk [8]. Furthermore, given that muscular fitness can be improved through structured resistance and functional training programs, it represents a practical and impactful intervention target in both school-based and community health initiatives aimed at preventing noncommunicable diseases across the lifespan.

Another relevant observation from our results is the relatively low correlation between each fitness variable and the cardiometabolic risk classification, with coefficients ranging from −0.06 to −0.12. This indicates that the relationship between physical fitness and cardiometabolic risk may not follow a linear pattern, which reinforces the decision to employ nonlinear machine learning models such as GB. The fact that simple correlations were weak while the model’s predictive performance was acceptable suggests that interactions and complex dependencies between variables contribute meaningfully to risk classification. This highlights the added value of advanced analytical techniques in capturing multidimensional health phenomena that traditional statistical methods may overlook. Furthermore, the absence of strong collinearity among the predictors suggests that each fitness test contributed unique and nonredundant information to the model, supporting the inclusion of all three components, VO_2max_, horizontal jumps, and push-ups, in the final classification algorithm.

Indeed, when a complex model such as GB performs well despite weak linear correlations, there is a legitimate concern that it may be capturing noise or specific idiosyncrasies of the training data, rather than learning generalizable patterns. This is a known limitation of high-capacity models in low-signal scenarios. In our exploratory analysis, the SHAP interpretability framework played a critical role in assessing whether the model relied on clinically plausible and domain-relevant features. By examining the direction and magnitude of each predictor’s contribution, we could evaluate whether the model’s decisions aligned with expert knowledge. Although this does not eliminate the possibility of overfitting, it supports the idea that the model was not purely exploiting noise but identifying meaningful interactions worthy of further investigation. This interpretability-centered validation reinforces the potential of the dataset and the modeling approach, even if the current model is not the final deployable version.

Our findings also align with growing evidence supporting the utility of WHtR as a reliable marker of central adiposity and early cardiometabolic risk in youth populations. For instance, Ashwell and Hsieh [45] emphasized that WHtR outperforms BMI in predicting health risks across various age groups, including children and adolescents, due to its better representation of visceral fat distribution. Moreover, Brambilla et al. [46] found that WHtR had a stronger association with cardiometabolic risk factors compared to BMI or waist circumference alone in school-aged populations. In the context of our study, the use of WHtR ≥ 0.5 as a threshold allowed for a practical and meaningful dichotomization of risk, compatible with international standards [48]. Although our model relied solely on this anthropometric indicator as the reference for cardiometabolic risk classification, the high classification accuracy achieved suggests that even basic measurements, when combined with physical fitness data and machine learning techniques, can provide robust screening tools for early risk detection.

In this context, a systematic review by Lima et al. [54] reported that muscular fitness, assessed by maximal muscular strength/power or muscular endurance, is potentially associated with lower levels of obesity and improved cardiometabolic health. However, there is limited support for an inverse association between muscular fitness and blood pressure, lipids, glucose homeostasis biomarkers, and inflammatory markers in children and adolescents. Moreover, our findings revealed that VO_2max_ had a low contribution as a predictor of cardiometabolic risk. However, most studies have focused on cardiorespiratory fitness as a predictor of cardiometabolic risk [55,56,57], but different studies have shown an inverse association of both muscular and cardiorespiratory fitness with the risk of metabolic syndrome in children and adolescents [58,59,60]. This implies that exercise-based interventions combining aerobic and resistance training are effective strategies for improving metabolic health in adolescents with excess weight. In particular, recent evidence from a systematic review and network meta-analysis by García-Hermoso et al. [61] demonstrated that high-intensity interval training (HIIT), especially when combined with resistance training, produced the greatest reductions in insulin resistance markers such as fasting insulin and homeostatic model assessment for insulin resistance (HOMA-IR) in children and adolescents with overweight or obesity. This study further identified a nonlinear dose–response relationship, showing that a minimum of 900 to 1200 metabolic equivalent task minutes per week (equivalent to two to three 60 min sessions of moderate to vigorous activity) was sufficient to achieve clinically meaningful improvements. These findings reinforce previous conclusions reported by Liu et al. [62], García-Hermoso et al. [61], and Mendelson et al. [63], underscoring the importance of structured and combined exercise protocols in reducing cardiometabolic risk during adolescence.

Currently, there are no studies that predict cardiometabolic risk based on physical fitness in adolescents; rather, different studies predict cardiovascular risk from different health indicators, as Salah and Srinivas [38] developed a machine learning-based explanatory framework for predicting long-term CVD risk (low vs. high) among adolescents through relevant survey questionnaires and health tests from adolescence to young adulthood. While all the machine learning models demonstrated good predictive ability, XGBoost performed the best, as in our study. The results of this study suggest that machine learning can be used to detect CVD in adulthood at very early stages of life. However, this study did not consider fitness variables to predict cardiometabolic risk. On the other hand, Musleh et al. [64] used various machine learning techniques by introducing a new feature of the ‘risk level’ derived through fuzzy logic applied to the Conicity Index. In this study, LR emerged as the best performer among men, achieving high-risk prediction. Both the SVM and LR lead to higher risk prediction performance among women.

### 4.1. Practical Applications

The results of the present study can be applied in school and public health contexts for the development of automated screening systems for cardiometabolic risk in adolescents based on simple and accessible physical tests. The implementation of machine learning models, such as GB, would allow for early identification of at-risk students, facilitating personalized preventive interventions that promote active and healthy lifestyles. This strategy could complement current approaches focused on anthropometric variables and expand the usefulness of data collected by educational systems for public health purposes. The findings of our study, after validation in new national or Latin American databases, could be used to develop cardiometabolic disease prevention programs through the promotion of healthy lifestyles, especially physical activity habits that could lead to a higher cardiorespiratory fitness, which is inversely associated with CVR in pediatric populations [65], as well as muscle strength [66], which allows for early diagnosis of cardiometabolic risk [49], opening up the range of risk factors that are limited to anthropometric variables that do not consider more holistic algorithms that combine different measures of body composition and lifestyle factors, including physical condition variables, physical activity habits, and sedentary time.

### 4.2. Limitations and Future Research Lines

This study has several limitations that should be acknowledged. First, due to its cross-sectional design, causal inferences cannot be made, and the directionality of the observed associations between physical fitness and cardiometabolic risk remains undetermined. Second, while the study incorporated multiple components of physical fitness, it was limited to two muscular fitness assessments (push-up and horizontal jump testing) and only one measure of cardiorespiratory fitness (i.e., VO_2max_). This imbalance in the number of tests may have introduced an implicit weighting favoring muscular fitness in the machine learning models. The absence of a standardized method to balance the influence of each fitness component may affect the interpretation of their relative predictive power. Third, other important dimensions of physical fitness, such as flexibility, speed, and agility, were not assessed, potentially overlooking additional relevant predictors of cardiometabolic health. Furthermore, although the WHtR is a widely validated proxy for central adiposity, it does not capture the full spectrum of metabolic risk factors, such as lipid profiles, insulin resistance, or inflammatory biomarkers. Lastly, external factors such as test administration conditions, participant motivation, and health status on the day of testing may have influenced performance outcomes, introducing potential measurement variability.

It is important to note that the AUC-ROC obtained by the GB model was 0.601, which corresponds to low discriminatory power according to conventional criteria. This result indicates that, although the model performed well on other metrics (accuracy, recall, and F1 score), its ability to correctly differentiate between adolescents with and without cardiometabolic risk is limited when evaluated solely by AUC-ROC. This limitation may be due, in part, to the simplicity of the set of predictors used (only three physical variables plus age and sex), as well as the use of a single reference indicator (WHtR). Future research should incorporate additional clinical biomarkers, as well as more dimensions of physical and behavioral fitness, to improve the overall discriminatory power of predictive models.

Future studies should adopt longitudinal designs to explore causal relationships between different components of physical fitness and cardiometabolic risk over time. It would also be valuable to include a broader range of fitness domains, such as flexibility, speed, and agility, to capture the multidimensional nature of physical fitness more accurately. Moreover, incorporating additional metabolic biomarkers (e.g., lipid profiles, inflammatory markers, and insulin sensitivity) could enhance the predictive capacity of machine learning models. Finally, efforts should be made to validate these predictive models in diverse populations and socioeconomic contexts across Latin America, ensuring their generalizability and applicability in real-world settings.

These findings highlight the potential of artificial intelligence as support in the design of health-oriented educational policies, facilitating the early detection of risk in the school environment and promoting the implementation of targeted physical activity programs that contribute to improving quality of life and preventing chronic diseases from early stages. However, before considering its implementation in real-world scenarios, the models must be validated in external and independent samples, both nationally and in other Latin American countries. This validation is indispensable to ensure its robustness, generalization, and practical utility in diverse contexts, especially those with limited access to clinical or laboratory infrastructure. Furthermore, the results of the model indicate that anthropometric data, particularly WHtR, remain an essential component for cardiometabolic risk classification. Their high contribution to the prediction model reinforces their value as a proxy indicator of central adiposity. This suggests that they should be considered a key variable in future screening and prevention strategies at the school and community level.

## 5. Conclusions

Among all the machine learning models used, GB stands out as the most effective tool for predicting cardiometabolic risk in Chilean adolescents based on physical condition variables, showing a solid balance between accuracy, sensitivity, and discrimination capacity. Its interpretability, ensured through techniques such as SHAP, allows for a clear understanding of the contribution of each variable to risk, which makes it a reliable resource for decision-making. In summary, our findings demonstrate the potential of artificial inteligence/machine learning models as supportive tools for early detection of cardiometabolic risk and for informing health-oriented educational policies.

## Figures and Tables

**Table 1:** Presentation of the most relevant default hyperparameters for each model used.

Algorithm	Key Parameter	Default Value	Brief Description
Gradient boosting	n_estimators	100	Number of trees (boosting stages).
	learning_rate	0.1	Weighting of each tree’s contribution.
	max_depth	3	Maximum depth of each tree.
Logistic regression	penalty	‘l2’	Type of regularization (Ridge).
	C	1.0	Inverse of the regularization strength.
K-nearest neighbors	n_neighbors	5	Number of neighbors to consider.
	weights	‘uniform’	All neighbors have the same weight.
Support vector ma-chine (linear support vector classifier)	kernel	‘rbf’	Radial basis kernel for nonlinear relationships.
	C	1.0	Regularization parameter.
	gamma	‘scale’	Kernel coefficient.
Random forest	n_estimators	100	Number of trees in the forest.
	criterion	‘gini’	Function to measure the quality of a split.
	max_depth	None	Nodes are expanded until they are pure.

**Table 2:** Descriptive characteristics of the sample.

Variables	Males (n = 4356)		Females (n = 3498)		All (N = 7854)	
	Level 1 (n = 3417)	Level 2 (n = 939)	Level 1 (n = 2659)	Level 2 (n = 839)	Level 1 (n = 6076)	Level 2 (n = 1778)
VO_2max_ (mL/kg/min)	28.8 (27.4–30.8)	27.4 (27.2–29.4)	28.4 (27.4–29.4)	27.4 (26.8–29.0)	28.9 (27.4–29.6)	27.4 (26.4–28.9)
Horizontal jump (cm)	165.5 (146–184)	152.7 (136.5–171)	125.8 (109.5–142.0)	118.8 (102.0–134.5)	147.0 (124.0–172.0)	136.5 (115.0–158.5)
Push-ups (reps)	16.0 (10–22)	13.7 (6–19.5)	15.5 (10.0–20.0)	13.0 (10.0–20.0)	15.0 (10.0–21.0)	13.0 (9.0–19.0)
WHtR	0.430 (0.410–0.450)	0.530 (0.510–0.570)	0.430 (0.410–0.460)	0.540 (0.520–0.560)	0.430 (0.410–0.460)	0.530 (0.510–0.560)

Data expressed as median (interquartile range). Level 1: Adolescents classified as having no cardiometabolic risk (i.e., WHtR < 0.5); Level 2 includes those at cardiometabolic risk (i.e., WHtR ≥ 0.5). VO_2max_: estimated maximal oxygen consumption; WHtR: waist-to-height ratio.

**Table 3:** Comparison of classification algorithms.

Algorithm	Accuracy	Error	Precision	Recall	F1 Score	AUC-ROC	Training Time (s)	Classification
Gradient boosting	0.770	0.229	0.597	0.770	0.673	0.601	0.295	Good performance
Logistic regression	0.773	0.226	0.598	0.773	0.674	0.595	0.012	Good performance
K-nearest neighbors	0.741	0.258	0.679	0.741	0.697	0.548	0.004	Good performance
Support vector machine	0.773	0.226	0.598	0.773	0.674	0.535	8.609	Good performance
Random forest	0.708	0.291	0.665	0.708	0.682	0.529	0.449	Good performance

Comparison of classification algorithms based on key performance metrics. Accuracy = proportion of correct predictions; error = 1 − accuracy; precision = proportion of true positives among predicted positives; recall = proportion of true positives among actual positives (also known as sensitivity); F1 score = harmonic mean of precision and recall; AUC-ROC = area under the receiver operating characteristic curve, representing overall model discrimination ability. Training time = time required to train the model in seconds. Performance classification indicates overall evaluation based on metric balance. All models were trained and tested using the same dataset.

## Data Availability

Data will be made available on request (link: https://informacionestadistica.agenciaeducacion.cl/#/bases (Accessed on 3 March 2025).
